# Revised classification of kinases based on bioactivity data: the importance of data density and choice of visualization

**DOI:** 10.1186/1758-2946-5-S1-P24

**Published:** 2013-03-22

**Authors:** Shardul Paricharak, Tom Klenka, Martin Augustin, Umesh A Patel, Andreas Bender

**Affiliations:** 1 Unilever Centre for Molecular Informatics, Department of Chemistry, University of Cambridge, Cambridge, CB2 1EW, UK; 2 Merck Millipore, Dundee Technology Park, DD2 1SW, Dundee, UK; 3 EMD Millipore Corporation, 15 Research Park Drive, St. Charles, Missouri 63304, USA

## 

Kinases are a major class of drug targets and are involved in a variety of diseases such as diabetes, cancer and inflammation. Still, understanding kinase inhibitor selectivity and promiscuity remains a major challenge. In order to improve upon the current situation, we analyzed a dataset comprising 157 compounds, tested at concentrations of 1 μM and 10 μM against a panel of 225 human protein kinases. Our bioactivity-based classification of kinases shows similarities with the Sugen sequence-based classification [1], where particularly kinases from the TK, CDK, CLK and AGC groups cluster together. However, 57% of all kinase pairs inhibited by 6 known inhibitors consist of kinases which lie far apart from each other in the Sugen tree (relative distance of 0.6 - 0.8 on a scale from 0 to 1), but are correctly located closer to each other in our bioactivity-based tree (distance 0 - 0.4). For 80% of all analyzed kinases, those classified as neighbors according to the bioactivity-based classification also show high similarity in shared active compounds. However, among the remaining ~20%, distant kinases did not necessarily show low SAR similarity, and neighboring kinases did not necessarily show high SAR similarity; i.e., the placement in the tree was misleading. We identified two reasons for this: firstly, 'misplaced' kinases exhibit inconsistent SAR, and secondly, these kinases had only a few shared activities with other kinases, making both the computation of their bioactivity-based distance and their place in the tree less accurate. In a follow-up analysis, we resolved both problems by visualizing inconsistent SAR more accurately using MDS plots, rather than phylogenetic trees, and by excluding kinases with 16 or fewer shared activities. Only 7 kinases (4% of the kinases analyzed) did not show a clear relationship between kinase bioactivity profile similarity and shared active compounds. Hence, this analysis improves on previous studies, where the influence of data density on kinase similarity was not considered, and leads to a more reliable placement of kinases into the kinome tree. Overall, our analysis suggests that bioactivity-based classification of kinases is indeed more useful than sequence-based classification for predicting kinase-inhibitor interactions. However, care needs to be taken with respect to data density (i.e., kinases with too few data points need to be omitted) and visualization of the data (i.e., phylogenetic trees imply a neighborhood relationship that is not consistently observed in every case).
